# Global Health – emergence, hegemonic trends and biomedical reductionism

**DOI:** 10.1186/s12992-020-00573-4

**Published:** 2020-05-06

**Authors:** Jens Holst

**Affiliations:** Department of Nursing and Health Sciences, Fulda University of Applied Sciences, Leipziger Strasse 123, D-36037 Fulda, Germany

**Keywords:** Global health, International health, Social determinants, Governance, Globalisation, Global burden of diseases, Health-in-all policies, Inequality, Hegemony, Decolonisation

## Abstract

**Background:**

Global Health has increasingly gained international visibility and prominence. First and foremost, the spread of cross-border infectious disease arouses a great deal of media and public interest, just as it drives research priorities of faculty and academic programmes. At the same time, Global Health has become a major area of philanthropic action. Despite the importance it has acquired over the last two decades, the complex collective term “Global Health” still lacks a uniform use today.

**Objectives:**

The objective of this paper is to present the existing definitions of Global Health, and analyse their meaning and implications. The paper emphasises that the term “Global Health” goes beyond the territorial meaning of “global”, connects local and global, and refers to an explicitly political concept. Global Health regards health as a rights-based, universal good; it takes into account social inequalities, power asymmetries, the uneven distribution of resources and governance challenges. Thus, it represents the necessary continuance of Public Health in the face of diverse and ubiquitous global challenges. A growing number of international players, however, focus on public-private partnerships and privatisation and tend to promote biomedical reductionism through predominantly technological solutions. Moreover, the predominant Global Health concept reflects the inherited hegemony of the Global North. It takes insufficient account of the global burden of disease, which is mainly characterised by non-communicable conditions, and the underlying social determinants of health.

**Conclusions:**

Beyond resilience and epidemiological preparedness for preventing cross-border disease threats, Global Health must focus on the social, economic and political determinants of health. Biomedical and technocratic reductionism might be justified in times of acute health crises but entails the risk of selective access to health care. Consistent health-in-all policies are required for ensuring Health for All and sustainably reducing health inequalities within and among countries. Global Health must first and foremost pursue the enforcement of the universal right to health and contribute to overcoming global hegemony.

## Background

Global Health is currently high on the international political agenda and plays an important role at summit meetings of international forums such as the “Group of 7” (G7) and the “Group of 20” (G20). The increasing political importance of Global Health and the consideration of this topic on the international stage is long overdue from the point of view of health sciences and health policy. The currently prevailing understanding of Global Health, however, exhibits some conceptual limitations as the scope and content of the respective debate is often inappropriate in capturing the full complexity of the challenges. The current Global Health discourse often fails to fulfil the claim to universalism implicitly associated with the term “global”. Moreover, it tends to neglect the requirements of a comprehensive transdisciplinary and interdisciplinary understanding of health policy. In fact, there is a large discrepancy between the current state of knowledge and Global Health policy practice [1].

In most countries around the world, health policy is primarily concerned with the inherent challenges of national health systems and puts the spotlight on health-financing reforms, universal health coverage, access to health care in rural areas and other local or regional challenges. However, public awareness of how global health has become in the meantime is regularly raised when a threat in the form of a potentially dangerous infectious disease appears [2]. When deadly infections hit the headlines, cross-border, international and increasingly global health problems attract the attention of people in the Global North. However, the succession of life-threatening scenarios caused by “killer viruses” and other epidemics that have long been considered defeated or at least controllable in high-income countries has become denser in recent years. What began with the spread of the AIDS pandemic has further developed in increasingly close timely order with the emergence of dangerous infectious diseases such as SARS (Severe Acute Respiratory Syndrome) in Southeast Asia in 2002 and swine flu in Northern hemisphere winter time 2009–2010, MERS (Middle-East Respiratory Syndrome) in 2012, and avian influenza from 2013 onwards. Particular attention was paid to the 2014 Ebola outbreak in Western Africa, which claimed more than 11,000 lives, and 5 years later in Eastern Democratic Republic of Congo, the Zika virus in Brazil, and most recently the coronavirus pandemic that originated in the Chinese province of Wuhan and spread around the world.

Particularly in Europe and North America, but also in Latin America and other emerging regions of the world, this wave of ever more successive epidemic outbreaks defined as ‘health crises’ repeatedly provoke a state of alert and make the headlines. However, public interest in the health-related challenges of other countries and continents is usually short-lived and transient. That makes a crucial difference in the low- and middle-income countries of the Global South and especially in Sub-Saharan Africa, where certain health hazards persist. In low-income countries and particularly among the poorest populations, infectious diseases still represent a relevant health threat and the risk of endemic diseases or even epidemics is part of everyday life.

However, today infectious diseases do not present the only challenge for people and health systems in developing countries. In the course of epidemiological transition, the disease spectrum is expanding from infectious to non-communicable, chronic diseases. This hitherto double burden of disease, caused by bacterial, viral or other pathogens on the one hand and health problems commonly referred to as chronic or civilisation diseases on the other, has increasingly burdened most developing countries and countries in transition during the last decades [3]. The simultaneous coexistence of undernourishment and malnutrition and dietary overweight exacerbates the situation [4].

### Emergence of Global Health

#### Motives and history of Global Health

Notwithstanding the little influence on the national health-policy debates within countries, Global Health has become one of the most important areas of foreign, development and security policy in the past 15 years [5]. Security is frequently encountered as contextual framework in political health and foreign-policy documents, and the securitisation of health is meanwhile considered a key feature of public health governance [6]. The rapid succession of endemic and epidemic outbreaks perceived as health crises has ultimately contributed to pave the way of health into international relations [7] and shape the securitisation of Global Health promoted by multiple agents at national and international levels who interact to target cross-border threats to health [8]. In fact, acute epidemic outbreaks are often seen to be a symptom of globalisation, while Global Health tends to ignore and conceal long-term diseases like tuberculosis and the structural causes of bad health and health inequalities [9]. The increasing international and political relevance of Global Health calls for more comprehensive governance strategies of institutions and processes, which have an explicit health mandate (Global Health governance), for institutions and processes of global governance which have a direct and indirect impact on health (global governance for health), and for national and regional institutions and mechanisms which are established for contributing to governing Global Health governance (governance for Global Health) [10].

Regardless of these collateral subjects, the concept of “Global Health” itself includes a broad array of subjects such as political approaches, research, teaching and clinical practice, and aims to improve health care and the necessary access to it as well as people’s health worldwide. Global Health encompasses both individual clinical care and prevention at the level of populations or people in the meaning of Public Health. Despite the diversity and heterogeneity of the definitions and actors involved, the concept also implies examining transnational contexts as well as the social, political and economic determinants of health and finding solutions to existing health problems. The understanding of Global Health ranges from health as an instrument of internal security and foreign policy to charitable philanthropies, public-private partnerships, general human rights and solidarity [11].

From the outset, International Health and Global Health were inextricably linked to both the protection of national populations and commercial interests and aspirations. For example, the US Institute of Medicine emphasised the protection of citizens in the United States of America (USA); they bluntly asserted that four of the world’s ten leading pharmaceutical manufacturers control 40% of the world market and that the introduction of new drugs and vaccines in developing countries offers the pharmaceutical and vaccine industry in industrialised countries good sales opportunities [12]. In its first Global Health Strategy, [13] the German government put a strong focus on the protection of the population of the Federal Republic of Germany and the economic interests of Germany’s export-oriented economy [14]. The German Ministry of Education and Research has so far concentrated research funding on neglected and poverty-driven diseases and is only gradually expanding the range of topics in the context of Global Health sciences [15].

An analysis of relevant publications shows the comparatively short history of the term “Global Health”, first in the scientific community and also later in the political debate. The use of Global Health in the English literature began in the 1990s, increased sharply since 2000, and at the beginning of the millennium overtook the hitherto prevailing use of the term “International Health”. The development in French and Spanish publications was comparable, although the alternative terms “mondial” and “mundial” had already been used earlier in relation with health [16].

However, the concept of Global Health did not fall from the sky but was instead developed from various predecessors that began in the “colonial medicine” of the 19th and early 20th centuries, which developed into “Tropical Medicine” and thereafter “International Health”. Since then, high-income countries have pushed the development of an international regime of infectious disease control, mostly driven by their own security interests. The International Sanitary Conference held as early as 1851 is generally considered to be the starting point for international cooperation in health [17, 18]. The primary focus on harmonising quarantine requirements in the European colonial power nations made the conference a crucial step towards international health security concerns. Until today, Global Health is often seen in the context of foreign policy and closely linked to international and health security.

In recent decades some paradigm shifts occurred in relation to international aspects and features of health [19]. Initially, the main focus was on preserving the health of European colonial rulers and protecting them from the health hazards of tropical diseases. From this, in close connection with the fields of “Hygiene” and “Public Health”, the predominantly clinical field of “Tropical Medicine” developed [20]. At the beginning of the twentieth century, terms such as “Medicine in the Tropics” and “Tropical Hygiene” were in the foreground. In this context, tropical institutes emerged on the European continent, which in the important port cities such as Antwerp, Hamburg and London mainly took care of seafarers landing on the coast and performed epidemiological hygiene tasks inland, some of which later came under the responsibility of the Public Health Services [21].

The view prevailing at that time, which is ultimately still valid today at least for powerful approaches to Global Health, was aptly described by the British pathologist and bacteriologist Harold Scott 80 years ago in his analysis of the historical development of tropical medicine: “We can then trace how improvements have been brought about, usually first with a view to safeguarding the health of officials and European traders, and later undertaking also the treatment of natives by which two purposes would be simultaneously accomplished - benefit to the health and well-being of the native and further protection of the white man from native-born infection.” [22]

### The origin of Global Health

In the second half of the twentieth century, and especially during the Cold War, the concept of International Health, a comparatively straightforward further development of traditional tropical medicine, became increasingly accepted [23]. International Health is mainly concerned with health problems and challenges in low-income countries. The main focus here is on measures to prevent and treat infectious diseases, to improve hygiene and water supply, and to promote child and maternal health [24]. Many universities and other scientific institutions still use this term until today, but with a broader understanding that also includes topics such as non-communicable diseases, injuries and the strengthening of health systems beyond tropical diseases.

In addition to health-related challenges in developing countries, International Health also refers to the commitment of high-income industrialised countries and the international organisations supported predominantly by them [25]. The emergence of development aid, the rather paternalistic predecessor of later overseas development aid and current international cooperation, also included helping low-income countries to overcome their health problems.

At about the same time, the Public Health concept, which had been further developed mainly in the United States of America and the United Kingdom after World War II, became increasingly important. Public Health developed from social hygiene or social epidemiology and exhibits some important differences from the individualised medical approach and the risk factor model. In contrast to the so-called disease sciences with their focus on individual health problems, Public Health and Health Sciences are explicitly population centred. Public Health is primarily concerned with the social determinants of health and illness as well as health inequalities due to the unequal distribution of social, political and economic opportunities.

### Global or planetary health

In the last two decades, the expanded and broader concept of “Global Health” has become established [26]. In the largely globalised world of the twenty-first century, population health is influenced by numerous factors that transcend national borders, ranging from pandemics to patents on drugs and climate change. With the shift in the worldwide health burden from infectious to non-communicable diseases (NCDs), the effects of lifestyles and other environmental determinants on people’s health have also come to the forefront. Global Health is not limited to cross-border health problems in the narrower sense. Rather, “global” in this context refers to every health challenge or transnational determinant, including the worldwide eradication of diseases (e. g. polio), antibiotic resistance, food security, urbanisation, migration and climate change [19].

Even broader and more comprehensive is the Planetary Health concept, which has only been noticed by the scientific community for a few years but was already discussed in the 1970s, and which explicitly considers the health effects of human activities on life in the biosphere [27]. “Planetary Health” corresponds to an attitude and philosophy towards life, focuses on people and not on diseases, and deals with the reduction of health inequalities due to income, education, gender and living environment with the objective to enable all people on the globe to enjoy the right to health and well-being, [28, 29] in order to “leave no one behind” [30]. The focus lies on the impact of environmental changes on human health. Planetary Health emphasises human health in the Anthropocene and the threats posed to the human species by pandemics or climate change, the natural spaces in which these species develop, and the health and diversity of the biosphere [31].

### Global Health as a component of globalisation

Towards the end of the last century, the dynamic trend towards increasing international interdependence in important areas of life such as politics, economy, culture and environment, which is generally referred to as globalisation, clearly picked up speed. The main prerequisites and causes for globalisation were technical progress due to product and process innovations, above all in communication and transport through the spread of the Internet and the significant increase in worldwide air traffic as well as flexible and more efficient means of transporting goods and services. The internationalisation and liberalisation of production and trade, increasing digitisation, new means of communication, growing migration pressure due to population growth, protracted conflicts and ecological challenges have further promoted and accelerated this globalisation.

### Growing global health burden

The global burden of disease is increasingly influenced by the conditions and effects of globalisation, including the worldwide dissemination of both infectious and non-infectious public health risks. Infectious diseases are primarily concentrated in rural areas of Sub-Saharan Africa, Asia, and Latin America. Regional variations exist in the distribution of these diseases as they disproportionately affect the poorest populations and contribute to a cycle of poverty due to decreased productivity [32]. Although the burden of disease in the poorest regions of the Global South continues to be determined by infectious diseases such as malaria, tuberculosis, and HIV, countries in this region are simultaneously undergoing a rapid epidemiological transition characterised by a shift from disease-burden profiles dominated by communicable diseases and childhood illnesses to profiles featuring an increasing predominance of chronic, non-communicable diseases along with accidents and injuries [33].

The often strong changes in daily life associated with globalisation have induced discernible and tangible health consequences in practically all countries around the world. The acceleration of everyday life, for example, increases the pressure on many people to perform, creates stress and exposes many gainfully employed persons to major direct and indirect risks. Today’s increasing health risks in the Global South are closely related to urbanisation and altered lifestyles, especially air pollution, unhealthy diet, physical inactivity, smoking, and excessive alcohol use [34]. The changes in working and living habits and their consequences for physical, mental and social health contribute to the global harmonisation of the disease spectrum, which in many developing and emerging countries is associated with a double burden of disease due to the simultaneous occurrence of infectious and non-communicable diseases [35, 36].

The globalisation effects observed in the Global North are likely to differ from those in middle- and low-income countries. Notwithstanding, abundant evidence shows that existing inequalities within and between societies play a crucial role in determining the health status of a population or population groups [37]. Moreover, the association between socioeconomic position and health risk factors exhibits variations over time and between world regions. Nonetheless, there are strong associations between absolute income poverty and fundamental determinants of health such as malnutrition among children, lacking access to safe drinking water and sanitation, and exposure to indoor air pollution [38].

It is true that the increasing global importance of health issues and challenges becomes most evident when highly contagious, dramatic infectious diseases tend to spread across the globe and threaten the Global North. The recent coronavirus pandemic has once more highlighted the health-crisis related perception of Global Health. Notwithstanding the public worries and anxiety regularly provoked by outbreaks of “killer viruses” that arouse associations with threatening scenarios leading to the extinction of mankind, the so-called non-communicable or chronic diseases, which are usually associated with permanent or lifelong use of health services and the corresponding costs for affected persons and systems, are much more significant from an epidemiological point of view [39]. Nonetheless, global funding for non-communicable diseases is comparatively low and poorly coordinated, and many global players are calling for increased efforts against rare diseases rather than against chronic non-communicable diseases [32].

In this context, it has to be stressed that the unprecedented level of prosperity existing in the world does not prevent the inequality in accessing health services from increasing rather than decreasing. The extremely unequal distribution of both health problems and the global burden of disease on the one hand, and financial and other resources on the other, poses particular challenges for Global Health [40]. As a consequent and consistent further development and continuance of Public Health at the international level, Global Health addresses national, regional and international health issues, determinants and solutions in the various sectors directly or indirectly relevant to health, and at their interfaces. This requires interdisciplinary cooperation between politics, science and society as well as an analytical understanding of the complex interrelationships and transdisciplinary action. The concept of Global Health pursues a comprehensive, holistic, multi- or transdisciplinary and human rights-based approach. As a synthesis of Public Health, which lacks an international orientation, and International Health, which pursues a transnational approach but focuses more specifically on actual health sector policy, tropical medicine and development cooperation, the Global Health concept intrinsically includes health problems beyond the influence of individual states and pursues an explicitly political approach. Particular attention is paid to governance issues and challenges, i. e. the politically responsible guidance and rulemaking of governments or other relevant decision-makers in order to ensure the effective performance of the various actors in the health sector and other relevant fields in the public’s interest.

### Health for all

Global Health also includes the goal of achieving “health for all”, which the then 134 member states of the World Health Organization (WHO) agreed upon already over 40 years ago in Alma-Ata, Kazakhstan [41]. However, this goal has remained utopia until today, not least because self-proclaimed pragmatists were partly able to restrict the concept of primary health care that was adopted as a strategy at the time and focused on social justice and democratic participation, to cost-efficient - or profitable - medical interventions. ‘Selective Primary Health Care’ seemed to promise the solution for poverty-related diseases without having to deal with poverty as a structural condition for disease [42].

This way of thinking also determines the actions of influential global health actors today. Bill Gates, former Microsoft mogul and today the world’s largest funder of health projects in poor countries through the “Bill & Melinda Gates Foundation” run jointly with his wife, has his own idea in regard to the needs for achieving Global Health. As a prominent guest speaker at the 2005 World Health Assembly, the highest decision-making body of the WHO, he told the ministers and heads of government present: “But the world didn’t have to eradicate poverty to eradicate smallpox - and we don’t have to eradicate poverty before we eradicate malaria. We must produce and supply a vaccine - and the vaccine will save lives, improve health and reduce poverty” [43].

This statement illustrates the unbroken belief in the unlimited curative power of biomedicine. At the same time, it is also self-evident for one of the richest people in the world. Redistribution is the magic word that interested circles like to denigrate with the term “envy debate”. Poverty reduction strategies should not target “the poor”, as was treacherously said in development cooperation at the beginning of the century, [44] but the richest of the rich, i. e. the 1 % of the global population that owns more than half of the world’s disposable income and wealth. Of course, Bill Gates cannot be interested in this. Yet it is precisely socio-economic inequality that increases global poverty [45] and has a negative impact on public health in societies [46].

### Overcoming global inequality

Hence, global justice is and has to be a central element of Global Health. Health as a human right and as a public good is increasingly receding into the background, while economic interests and marketability are gaining importance. At present, social movements in many places play a stronger role in combating health rights than the state, although the latter is ultimately responsible for enforcing the right to health [47]. Therefore, the core objective of Global Health policy must be to reduce or even overcome inequalities that exist worldwide. This is closely linked to the Sustainable Development Goals (SDG) agreed upon by the international community in 2015 [48] and the measures to implement the Agenda 2030 [49]. The aim of the Agenda developed by governments with the participation of civil society around the world is global economic progress in harmony with social justice and within the framework of the Earth’s ecological limits. It is noteworthy that the Agenda and thus the SDGs claim to apply equally to all countries of the world - at least apart from such fundamental problems as hunger, poverty and mother-child mortality [50]. In contrast to the previous MDGs, it is no longer only the developing countries and countries in transition that are called upon to take action, but also the industrialised nations of the Global North.

Moreover, the SDG exhibit a clear focus on reducing inequalities within and among countries around the world through universal and comprehensive policies, which pay attention to the needs of disadvantaged and marginalised populations [48]. The SDG agenda challenges health policy-makers to identify a broader array of health policy and systems priorities than those associated with the former Millennium Development Goals (MDGs). The objective of the SDG is to stimulate multisectoral action through processes, policies, and programmes outside the health sector, that have health implications through social, commercial, economic, environmental, and political determinants of health [51].

### Less biomedicine, more public health

Common definitions make Global Health little more than an updated reprint of previous concepts. To this day, particularly medical, biotechnology and political actors regard Global Health primarily as a continuance of International Health and tend to marginalise the perspective of social medicine and social determinants of health [52]. Biomedical reductionism as promoted by major Global Health players including the WHO and Gates Foundation in relation to HIV, vaccination and other pharmaceutical solutions tends to supplant calls for more community health efforts [53]. This understanding is recognisably shaped by the legacy of colonialism and Western-dominated expertise on the “tropical” world and its challenges [54]. Thus, the prevalent concentration of Global Health policy on both cross-border health problems and the spread of dangerous infectious diseases often lacks an in-depth understanding of political, social and economic conditions and requirements. Policies and health strategies are often lacking the intrinsic complexity and universality of Global Health, most recently in the context of the coronavirus pandemic (see below).

There are certainly good opportunities for integrating political and technical approaches to communicable and non-communicable health problems [55, 56]. Effective surveillance and improved treatment options for infectious diseases can create synergies in preventing, screening and care of non-communicable diseases. There is some evidence suggesting that the experiences gathered from the scale-up of HIV/AIDS interventions can be successfully applied and adapted to the management of non-communicable diseases such as hypertension, diabetes mellitus and other chronic, non-infectious diseases [57–59]. Hence, the attempts to overcome the silos of vertical or stand-alone health programmes by expanding them to diseases and health problems they were not primarily designed for, were successful in some ways.

However, the claim for integrating approaches to communicable and non-communicable diseases by combining the implementation and improvement of surveillance with targeted health-in-all policy interventions, which are promising to be successful and cost-effective at the time, [34, 60] has not yet received a significant practical response. The latter include a broad array of measures such as restrictions on tobacco access and sales e. g. by sales bans for adolescents or raising tobacco taxes, limiting the use of alcohol and other harmful drugs, enacting speed limits, making the use of motorcycle helmets and seat belts legally binding, introducing legal requirements to reduce air and other environment pollution, and enforcing measures to reduce potentially hazardous components such as salt, sugar and trans fats in commercial food products, among others. The impact of these interventions, which are typically implemented at national levels, on communicable diseases is rather indirect and modest, and opportunities for integrating approaches are still to be found.

This is partly due to the fact that the integration of these types of interventions into Global Health practice is often taken for granted or, at best, not problematised as a legitimate political challenge [61]. Even more, approaches to implementing health-in-all policies are repeatedly surpassed by biomedical and technocratic approaches that tend to occupy the forefront of public interests and political decision-making. There are barely any Global Health documents that do not mention the social determinants of health; however, the adequate consideration and implementation is lagging behind the aspirations. One reason is that the healthcare sector is organised around special professional interests rather than prepared to deal with the contemporary public and Global Health challenges, which are all cross-cutting in nature and require intersectoral approaches [62]. The most prominent multilateral organisations at the forefront of tackling Global Health, such as the Global Fund to Fight AIDS, Tuberculosis and Malaria (GFATM), follow a vertical approach, put special emphasis on selected health challenges and use to apply instruments which are specific to a certain disease (e. g. HIV/AIDS in the case of the GFATM, or vaccine provision in the case of the Global Alliance for Vaccines and Immunzations - GAVI) or target group (e. g. children in the case of UNICEF). They have hardly contributed to health-systems strengthening [63] or funding [64]; nor have they pursued broader health-in-all strategies beyond improving health services and healthcare delivery. This imbalance is particularly reflected in Global Health initiatives such as GAVI dedicated to saving children’s lives and protecting people’s health by improving access to immunisation in poor countries. The general public around the globe is usually much less aware of the economic, social, political and other determinants of health than of the impact of worldwide epidemics on economic, social, political and other conditions of human life [65].

The current coronavirus pandemic has demonstrated the relevance of political and especially economic health determinants in a very different way from the perspective of Public Health and Global Health research. Today, health security is seen as a means for protecting the industry from the consequences of bad health rather than as a strategy for protecting people from the harmful impact of industry on their health. At the same time, the dichotomy between infectious diseases and health-in-all policies is becoming impressively evident in the pandemic. At the peak of the outbreak, the public and political debate is widely dominated by biomedical and biotechnical topics and restricted to the expertise of virologists and immunologists who form a kind of opinion monopoly that is able to determine political decision making as well as the reporting of the epidemic all over the world. The unceasing and massive coverage of coronavirus by policy and media pushes other policy issues and reporting to the margins, [66] only few non-mainstream journals analyse the pandemic in the context of social, political and economic determinants [67].

Likewise, the imbalance between biomedical and biotechnical approaches and strategies to influence the social determinants of health is reflected in the new megatrial launched by WHO for accelerating the research on medicines to fight the current coronavirus pandemic. The wish to obtain an efficient and safe therapy for the novel coronavirus is more than understandable but the results will only be short-lived and lose their value when a new virus appears. In spite of this and the crucial importance of non-medical factors, there is no comparable research fund yet in sight for investigating the social, political, economic and ecological determinants of the development and the overcoming of the pandemic. This is striking as the global spread of the virus and the very different regional and national strategies for combating it provide an ideal laboratory for comparative field research.

As important as good medical care is, it has less influence on people’s health than their living, working, environmental and other conditions. Without adequate consideration of the social determinants of health, the question of income and wealth, education, environment and other social factors, the health of the world’s population cannot be improved sustainably. This vision is lacking in many medical and health-science publications where technological measures prevail over strategies to eliminate and address underlying causes [68] or is incomplete in others [69]; and it has not yet found its place in the broader debate on Global Health. The German Platform for Global Health, an association of trade unions, non-governmental organisations and scientists, repeatedly points out the significance of the social conditions of health and the need to bring non-medical determinants more into the national and international health debate [70, 71]. In today’s globalised world, the main factors influencing people’s well-being and health can be controlled and influenced less and less at the national level alone. Nevertheless, the following also applies: Global Health is closely interlinked with national and local health issues [40].

For reducing the burden of both communicable and non-communicable diseases, interventions in the globalised economy and particularly in the global food industry are becoming more promising in regard to integrated approaches. There is growing evidence that the deadly epidemic outbreaks caused by different types of viruses during the last two decades are largely driven by anthropogenic changes, namely by human population density and especially by the expansion of agriculture and the global food industry [72, 73]. The constant loss of biodiversity and rapid deforestation raise the risk of these infections by bringing people and livestock into contact with wildlife, and by altering the environment to favour transmission of certain diseases, such as malaria, Ebola, Zika, dengue and coronaviruses causing severe acute respiratory syndromes [74–77].

In order to develop and implement an appropriate and effective Global Health policy, much more than biomedical, clinical or genetic engineering approaches are needed. Vertical programmes, which focus on restricted objectives and usually address only a certain part of the existing demand, besides other disadvantages, [78] or the development of new drugs and vaccines may be helpful, but they do not entail any changes to the underlying conditions and prevailing health problems of the world. Global Health policy must bring about a fundamental change in the understanding of health and take into account the complexity of health in all its breadth and diversity; it can only become effective when it is recognised as a cross-cutting issue in all policy areas and a health-in-all policy has become established. The focus on security issues and the consideration of Global Health policy as a means of preserving privileges and vested interests in an unequal world does not offer a solution to the existing challenges [79]. Global Health needs more health promotion than disease management; good work and income conditions for all; equal opportunities; the reduction of socio-economic and health inequalities; food sovereignty; responsible environmental policy; social security, peace, democracy and participation [80].

### Hegemony in Global Health

The historical roots of the predominant concept of Global Health go back to the period of European colonialism and are closely linked to the efforts of the colonial powers to secure their supremacy and interests in formerly dependent countries and regions. This hegemonic approach and claim to “Global Health” from the very beginning is still more or less evident today [81]. The unequal balance of power in times of politically and militarily enforced colonialism was more bluntly visible and ideologically covered by racial superiority, but Global Health reproduces the unequal relations and global inequalities until today. The scientific debate and research on Global Health is dominated by North American and European universities, which play a vital role in this field and sustain Global North-South research gaps [82, 83].

Likewise, the political debate is strongly influenced by the meetings of the Heads of State and Government in the G7 and G20, which, incidentally, are not international organisations and have no politically legitimate mandate beyond the existing power relations. The same applies to the philanthropic foundations which withhold taxes from the public budgets of the countries of the North and, due to their sheer financial strength in times of chronically emaciated public budgets, play a decisive role in determining the Global Health agenda, and tend to push through the privatisation of basic health and education services [84].

Moreover, Global Health policies are increasingly determined by foreign-policy priorities and security concerns. In 2014, more than 60 governments, international organisations and non-governmental stakeholders launched the Global Health Security Agenda (GHSA) as a concept to address the outbreaks of infectious diseases and reduce their spread to other countries [85]. Global Health security is often used to justify restrictive immigration policies and practices that restrict population movement across international borders by framing the migration of people as a risk. Rather than enhancing the local health system capacities, public policies in the name of Global Health security tend to focus on the protection of national borders in the Global North against perceived health threats from countries in the Global South [86]. However, it has to be pointed out that the fear-based focus on the prevention of and protection from infectious diseases is a clearly hegemonic approach that is far from adequately reflecting the global burden of disease, which is largely determined by non-communicable diseases [87]. In addition, the focus on health security often prevents or, at least, postpones the necessary debate about social, economic, and political determinants of health.

### Decolonising Global Health

There is growing criticism that Global Health is an unequal project in itself that carries forward the tradition of colonialism [81]. This is being reflected in the analysis of the manifold global partnerships in the fields of research and health care that have developed in recent years, especially between institutions in the North and those in the South. Such cooperation primarily benefits rich partners from high-income countries, as there is usually no appropriate political and social embedding of the results and successes in the systems of developing and emerging countries [88]. Often the funds provided by the richer partners flow past the national health systems or the projects even require additional funds that are then no longer available for the care of patients in rural areas or throughout the country [89]. Ultimately, many such partnerships reproduce global inequalities in access to and use of resources [90].

This has not been changed by ongoing globalisation or by the paradigm shift in development and international cooperation intended to be initiated through the Paris Declaration on Aid Effectiveness and the Accra Plan of Action [91]. Cooperation between institutions in high-income and poor and middle-income countries generally and almost inevitably involves a hierarchical relationship [83, 92]. Scientists from low-income countries are only gradually developing their own requirements and adapting their profiles for meaningful exchange with the Global North [93]. The relationship between institutions in North America, Europe and Australia, on the one hand, and research and care institutions in former colonial low-income countries, on the other, is often reflected in a cooperation that is regarded as ahistorical, apolitical and uncritical [94].

The connection between hegemony and inequality in Global Health is also reflected in the fact that most funding for Global Health projects comes from former colonial powers or philanthropic foundations. Given the global distribution, this is not surprising and can be well justified. The problem, however, is that the Global Health strategies that are carried and dominated by the rich part of the world reproduce precisely those processes that have led to their prosperity and thus to the extremely unequal global distribution of resources.

## Conclusions

In the globalised world, Global Health policy has become an important and complex cross-cutting issue. The fact that in recent years the global context of health has increasingly come to the fore is encouraging as long as Global Health is not reduced to epidemiological preparedness for preventing the cross-border spread of infectious diseases particularly to high-income countries. Biomedical and technocratic reductionism leads to selective access to health care, and privatisation increases rather than reduces health inequalities. Global Health has to emphasise the social, economic and political determinants of health. Health-in-all policies are required for ensuring Health for All and sustainably reducing health inequalities within and among countries. Global Health requires an understanding of human rights that does not regard health as a profitable “business model”, but as the aspiration of every human being. It must first and foremost pursue the enforcement of the universal right to health and contribute to overcoming global hegemony. Global Health must also address the causes of the impoverishment of the Global South, namely colonialism, the economic order oriented towards short-term profit maximisation and the ecological exploitation of natural resources in particular. Responsible Global Health policy has to address the causes of existing problems and must not limit itself to restoring the conditions that led to the global and planetary health crisis.

## Data Availability

Not applicable.

## Abbreviations

G7: Group of 7
G20: Group of 20
GAVI: Global Alliance for Vaccines and Immunzations
GFATM: Global Fund to Fight AIDS, Tuberculosis and Malaria
GHSA: Global Health Security Agenda
HIV: Human Immunodeficiency Virus
MDG: Millennium Development Goals
MERS: Middle East respiratory syndrome
NCD: Non-communicable diseases
SARS: Severe Acute Respiratory Syndrome
SDG: Sustainable Development Goals
UNICEF: United Nations International Children’s Emergency Fund
USA: United States of America
WHO: World Health Organization
